# Spontaneous Cure of Cancer of the Breast

**Published:** 1860-04

**Authors:** 


*Spontaneous Cure of Cancer of the Breast.*

Guerdan, of Billigheim, having a female under treatment for cancer of the left breast, with swelling of the axillary glands, sent her to Professor Chelius, who pronounced the case not suited for operation. It was decided then to employ the hemlock plaster. The physician was sent for, one evening, in great haste, and finding the patient bathed, as it were, in a pool of arterial blood, he ordered, without any great hopes as to the result, five drops of tinc. ferri. muriat. æther. every half hour. On his seeing her again, she told him that, after her return from the hospital, erysipelas had appeared on the diseased breast, which surrounded the tumor with a dark-red circle, for which she had employed fomentations of cold water. After some days, the circle changed its color from a bluish-red to a leaden hue; the scirrhous breast was covered with sanies; by degrees the whole diseased mass was decomposed into a granulated mass, analogous to a mixture of sanies and gluten, and, in five months, the whole cancerous breast was removed, leaving the pectoralis major exposed. Not only did granulations form a normal cicatrization, but the axillary glands, whose volume had diminished one-half during the suppuration, continued to disappear, until it was difficult to detect them by the touch. There remained nothing abnormal on the cicatrix, except a horny crust, which was kept covered with charpie and flannel. From that time, this person enjoyed good health, presenting no trace of cancerous disease or diathesis, and died eight years after, of an acute pleurisy.—*Echo Medical Suisse.* L. H. S.

